# A Huge Subcapsular Splenic Cyst Like Hematoma in Sickle Cell Anemia

**DOI:** 10.7759/cureus.22582

**Published:** 2022-02-24

**Authors:** Ahmad M Odeh, Kawthar A Boumarah, Wejdan A Alsumaien, Mohmmed T Al-Abbad, Aminah H Al-Ali, Zainab A Alammar, Hesham Alsuqair, Abdulqader M Albeladi, Abdulmohsen Alsuwaigh, Ammar Omrani, Mohammed M Almuhanna, Zaki Busbaih, Hussain R Al-Shaban, Abrar A Aldhameen

**Affiliations:** 1 General Surgery/Laparoscopic Surgery, Prince Saud Bin Jalawi Hospital, Al-Ahsa, SAU; 2 General Surgery, King Fahad University Hospital, Al-Khobar, SAU; 3 General Surgery, King Fahad Hospital, Al-Ahsa, SAU; 4 General Surgery, Prince Saud Bin Jalawi Hospital, Al-Ahsa, SAU; 5 Medicine, King Faisal University, Al-Ahsa, SAU; 6 General Surgery/Bariatric and Laparoscopic Surgery, Prince Saud Bin Jalawi Hospital, Al-Ahsa, SAU; 7 Laparoscopic Surgery, Prince Saud Bin Jalawi Hospital, Al-Ahsa, SAU

**Keywords:** cholecystectomy, splenectomy, sickle cell disease, sickle cell disease complications, splenic hematoma

## Abstract

Nontraumatic splenic rupture and hematoma are rare in sickle cell disease. We present a case of a 22-year-old Saudi male with sickle cell disease. He presented to our hospital with a history of nontraumatic abdominal pain, hemodynamic instability, and abdominal tenderness, with a large mass extending to the umbilicus. A computed tomography (CT) examination showed splenomegaly and a spleen infarction. The patient was admitted to the intensive care unit (ICU) and stabilized. He was transferred to the regular ward and discharged against medical advice (DAMA). Later on, he presented again with persistent abdominal pain. He underwent splenectomy with cholecystectomy. The patient did well postoperatively and was discharged in good condition. While conservative management is common, operative management should be considered in patient with persistent pain. Splenic rupture has a high mortality rate.

## Introduction

Sickle cell disease (SCD), a hereditary monogenic hemolytic anemia, is a public health issue in Sub-Saharan Africa, India, and the Middle East, especially Saudi Arabia [1]. The prevalence of SCD in Saudi Arabia differs significantly in different parts of the country; the highest prevalence is in the Eastern province, followed by the southwestern provinces. Although SCD is a monogenic disorder, patients have multiple systemic manifestations, ranging from anemia and pain crises to acute chest syndrome, hepatic sequestration, splenic sequestration, and stroke [2]. Splenic complications like hypersplenism, sequestration crisis, and infarction are common in patients with SCD [3]. SCD is the primary hemoglobinopathy worldwide in terms of frequency and social impact. Recently, the World Health Organization recognized SCD as a global public health problem [2]. Identifying new manifestations and complications plays a major role in decreasing the morbidity and mortality of SCD. Accordingly, we present a rarely documented complication of SCD identified as splenic hematoma-like cyst.

## Case presentation

**Figure 1:**
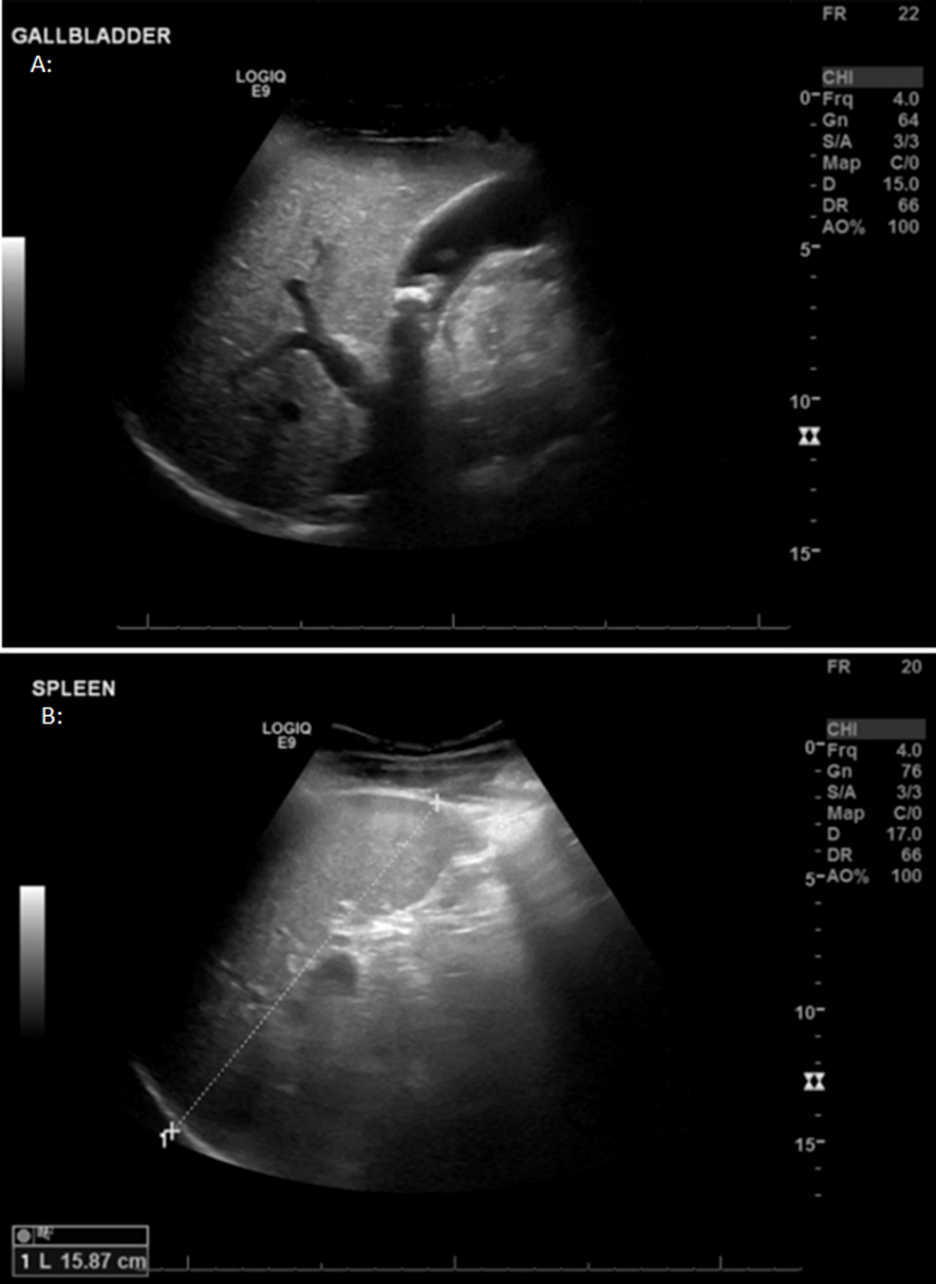
Abdominal ultrasound showing multiple gall bladder stones (A) and splenic enlargement measuring 15.9 cm (B).

The patient, a 22-year-old Saudi male with SCD, used to have recurrent vaso-occlusive crises, with a rate of one to two admissions annually. He presented to our hospital complaining of generalized body pain and fever for two days. On examination, the patient was conscious and alert; his vitals were as follows: the temperature was 38.2°C, pulse was 116 beats per minute (bpm), blood pressure was 90/50 mmHg, respiratory rate was 28 breaths per minute. His abdomen was distended with generalized tenderness and a palpable mass in the left upper quadrant extending to the level of the umbilicus, dull on percussion. Ultrasonography (US) revealed splenomegaly and gallstones (Figures 1A, 1B). 

**Figure 2:**
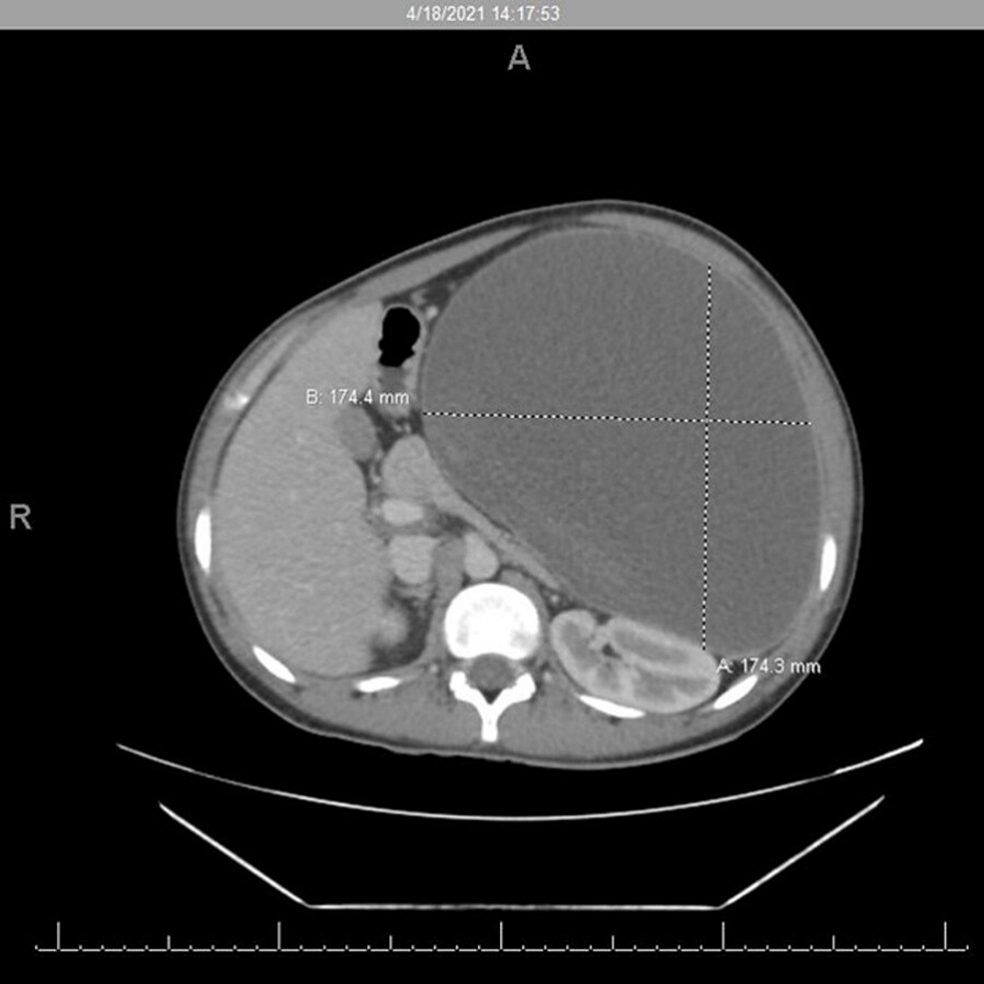
Abdomen CT scan showing spleen enlargement and a large subcapsular fluid collection.

The patient was admitted to the intensive care unit (ICU) for strict observation and stabilization, his hemoglobin (Hb) dropped from 11.8 g/dL to 7.7 g/dL. He received two units of blood transfusion. Computed tomography (CT) imaging showed an enlarged spleen, measuring 17.4 × 17.4 × 26.4 cm (AP × TR × CC), with inferior pole infarction associated with large necrotic fluid collection within the splenic capsule and multiple gallbladder stones (Figure 2). The patient’s symptoms persisted, and he developed severe abdominal pain, abdominal distension, nausea, vomiting, constipation, and oliguria. The patient moved to the surgical ward after seven days in the ICU.

During surgical ward admission, the patient was treated conservatively for a week with remarkable improvement of his symptoms, but he discharged himself against medical advice. Table 1 shows the patient’s lab results on the day of discharge. The patient was readmitted four months later with persistent symptom and 17 kg weight loss for preoperative evaluation. Three days before surgery, he had a transfusion of two units packed red blood cells based on hematology consultation.

**Table 1:** Laboratory investigations in the first ICU admission, third day in ICU admission, and the discharge day. WBC: white blood cell; Hb: hemoglobin; PLT: platelet; ALP: alkaline phosphatase; AST: aspartate aminotransferase; ALT: alanine transaminase; bili: bilirubin; K: potassium; Na: sodium; INR: international normalized ratio; PT: prothrombin time; PTT: partial prothrombin time; FBS: fasting blood glucose

Lab parameters	First ICU admission (March 21, 2021)	Third day in ICU (March 23, 2021)	On first discharge (April 4, 2021)	Normal values
WBC	17.8	30.66	16	4-10 × 10^9^/L
Hb	11.8	7.7	7.4	12-15 g/dL
PLT	268	47	830	130-400 × 10^9^
Albumin	36.6	39.6	30	30-50 g/dL
ALP	560	490	89	50-136 IU/L
AST	528	877.8	25.8	0-40 IU/L
ALT	70	601.90	51.3	30-65 IU/L
Total bili	72.2	57.1	24.5	0-24 mmol/L
Direct bili	7.05	11.3	9.9	0-5 mmol/L
FBS	7.4	7.73	4.41	4.1-8.3 mmol/L
K	3.11	6.52	4.41	3.4-5.1 mmol/L
Na	137	129	132	133-148 mmol/L
INR	1.95	1.57	-	0.9-1.2%
PT	22.9	18.4	-	9.8-13.2 s
PTT	49.8	58.2	-	26- 36 s

As the patient was young, symptomatic, unable to tolerate oral feeding, the decision was to take him for surgery under general anesthesia. On entering the abdominal cavity through a midline laparotomy incision, the lower pole of the cyst was at the level of the umbilicus, and it was adherent to the greater omentum. There were extensive adhesions surrounding the spleen and the cyst. The cyst was aspirated to decrease its size; it contained large quantities of necrotic cells.

**Figure 3:**
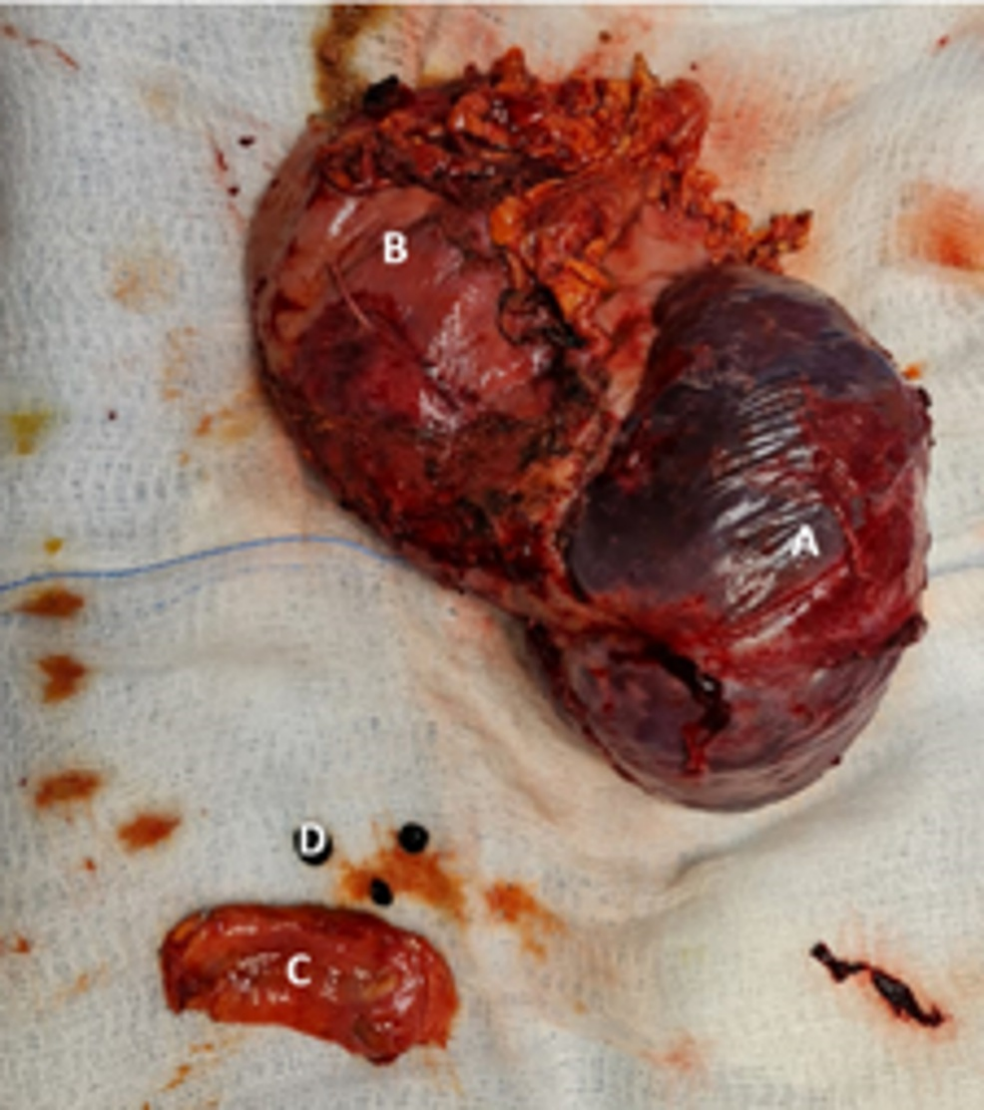
The image is showing surgically excised specimens - (A) spleen, (B) splenic cyst, (C) gall bladder, and (D) gall stones.

The patient underwent adhesiolysis around the cyst wall and spleen. The superior, posterior, and lateral aspects of the spleen were adhered to the patient’s diaphragm and surrounding organs, requiring blunt and sharp dissection by fingers and LigaSure (Dublin, Ireland: Covidien). The lesser omentum was opened for identification of the splenic artery on the upper border of the pancreas, where it was blighted and secured. The patient underwent dissection at the splenic hilum with multiple ligations and cuts in between. After securing all the supplying and draining vessels, the spleen was dissected and removed from the abdomen with the attached cyst (Figure 3). Besides that, the gall bladder was adherent to the surrounding organs. The patient underwent further adhesiolysis; the cystic duct and cystic artery were identified and clipped. The gallbladder was then removed from the abdomen.

After achieving completed homeostasis the abdominal cavity was washed and two drains were placed in the abdominal cavity, one at the splenic bed and the other at the pelvic cavity. The laparotomy incision was closed using one polydioxanone suture (PDS) and the skin was closed by stapler. The patient remained in the ICU for three days for close monitoring and was then transferred to the surgical ward for seven days. Pathology reports indicated splenic infarction, negative for malignancy, with chronic cholecystitis of the gallbladder. Cytological results of cystic fluid revealed cellular debris macrophages and RBCs, negative for malignant cells. On discharge, the patient was clinically well, in good condition, received full vaccinations, with stable vitals and normal labs (Table 2).

**Table 2:** Laboratory investigations in the elective admission for splenectomy and cholecystectomy. WBC: white blood cell; Hb: hemoglobin; PLT: platelet; ALP: alkaline phosphatase; AST: aspartate aminotransferase; ALT: alanine transaminase; bili: bilirubin; K: potassium; Na: sodium; INR: international normalized ratio; PT: prothrombin time; PTT: partial prothrombin time

Lab parameters	On first day of elective admission	On discharge	Normal values
WBC	10.9	10.92	4-10 × 10^9^/L
Hb	11.3	13	12-15 g/dL
PLT	320	597	130-400 × 10^9^
ALP	99	104	50-136 IU/L
AST	24.5	38.4	0-40 IU/L
ALT	22.3	50.8	30-65 IU/L
Total bili	82.6	51.7	0-24 mmol/L
Direct bili	10.6	14.3	0-5 mmol/L
Fasting glucose	5	3.95	4.1-8.3 mmol/L
K	4.52	4.79	3.4-5.1 mmol/L
Na	142	138	133-148 mmol/L
INR	1.4		0.9-1.2%
PT	13.3		9.8-13.2 s
PTT	42.5		26-36 s

## Discussion

Blunt abdominal trauma is the most common cause of splenic rupture and hematoma. However, there were reports of nontraumatic occurrences in patients with splenomegaly or underlying hematological abnormalities. Infectious pathologies; benign or malignant hemopathies; solid or cystic tumors; pancreatitis or other diseases with splenic tropisms, such as rheumatoid purpura, lupus, post-partum, or renal failure with dialysis; anticoagulation; or fibrinolytic treatment have also been reported [4]. Sickle cell anemia is a heredity hemolytic anemia that is an uncommon cause of spontaneous spleen rupture. It might be due to pathophysiological processes like sickle red blood cells with an uneven form and a proclivity for adhering to blood cells blocking blood vessels. This adherence causes congestive splenomegaly and splenic infarcts, making the spleen more vulnerable to rupture [5-7]. Our patient denied any history of trauma. His condition was caused by chronic splenic infarcts triggered hematoma formation, shown by the underlying sickle hemoglobinopathy and progressive abdominal pain. The patient’s hemoglobin dropped from 11 to 8, and CT imaging showed an enlarged spleen measuring 17.4 × 17.4 × 26.4 cm with infarction and large necrotic fluid collection contained within the splenic capsule. Finally, the pathological specimen revealed splenic infarction and RBC in cytology which complies with the diagnosis of chronic splenic infarction that progressed to subcapsular hematoma in the form of a huge cyst.

In some cases, the splenic hematoma progresses to splenic rupture. The splenic hematoma expanded in size in our patient until it became a huge subcapsular cyst-like hematoma without rupture. Contrast-enhanced CT is the imaging modality for diagnosing subcapsular hematoma, splenic infarct, pseudocyst, or for ruling out any other pathology [8]. In this case, the CT showed an enlarged spleen with an inferior pole infarction associated with a large necrotic fluid collection contained within the splenic capsule. The patient had multiple stones in the gallbladder. A subcapsular hematoma is the most common cause of delayed splenic rupture; however, it is not an indication for operative management of the injured spleen in a hemodynamically stable patient [7]. Conservative management is the mainstay treatment with observation and stabilization, including ICU care if needed. Nevertheless, the initial conservative management does not exclude the need for elective splenectomy later on, and it should not be delayed or neglected to avoid the risk of a future rupture due to the significant mortality rate linked with delayed spleen rupture diagnosis [7-9,10-12]. Our patient was presented with hemodynamic instability, with dropped Hb, associated with abdominal pain, distension, inability to tolerate oral feeding, the decision was to push for surgery after stabilization, blood transfusion, and imaging studies to prepare for surgery. Chronic splenic rupture is often accompanied by extensive peri splenic adhesions, making surgery difficult. Sharma reported a case similar to our case where surgical intervention was due to abdominal pain; he decided on a subcapsular splenectomy to avoid iatrogenic injury to surrounding organ structures [5]. Our patient had a successful splenectomy in the presence of dense tissues adhesion, in addition to cholecystectomy for chronic cholecystitis of pigmented stones.

## Conclusions

In conclusion, splenic rupture and the development of a huge subcapsular hematoma should be kept in mind as a differential diagnosis in sickle cell patients with nontraumatic acute abdominal pain. After diagnosing splenic subcapsular hematoma and stabilizing the patient, elective splenectomy should be planned, as the patient with subcapsular hematoma will stay at risk of splenic rupture, and to avoid morbidity and mortality for this complication, the surgical intervention should not be delayed.
